# Activation of pH-Sensing Receptor OGR1 (GPR68) Induces ER Stress Via the IRE1*α*/JNK Pathway in an Intestinal Epithelial Cell Model

**DOI:** 10.1038/s41598-020-57657-9

**Published:** 2020-01-29

**Authors:** Chiaki Maeyashiki, Hassan Melhem, Larissa Hering, Katharina Baebler, Jesus Cosin-Roger, Fabian Schefer, Bruce Weder, Martin Hausmann, Michael Scharl, Gerhard Rogler, Cheryl de Vallière, Pedro A. Ruiz

**Affiliations:** 1 Department of Gastroenterology and Hepatology, University Hospital Zurich, Zurich, Switzerland; 2 Zurich Center for Integrative Human Physiology, Zurich, Switzerland

**Keywords:** Transcription, Inflammatory bowel disease

## Abstract

Proton-sensing ovarian cancer G-protein coupled receptor (OGR1) plays an important role in pH homeostasis. Acidosis occurs at sites of intestinal inflammation and can induce endoplasmic reticulum (ER) stress and the unfolded protein response (UPR), an evolutionary mechanism that enables cells to cope with stressful conditions. ER stress activates autophagy, and both play important roles in gut homeostasis and contribute to the pathogenesis of inflammatory bowel disease (IBD). Using a human intestinal epithelial cell model, we investigated whether our previously observed protective effects of OGR1 deficiency in experimental colitis are associated with a differential regulation of ER stress, the UPR and autophagy. Caco-2 cells stably overexpressing OGR1 were subjected to an acidic pH shift. pH-dependent OGR1-mediated signalling led to a significant upregulation in the ER stress markers, binding immunoglobulin protein (BiP) and phospho-inositol required 1α (IRE1α), which was reversed by a novel OGR1 inhibitor and a c-Jun N-terminal kinase (JNK) inhibitor. Proton-activated OGR1-mediated signalling failed to induce apoptosis, but triggered accumulation of total microtubule-associated protein 1 A/1B-light chain 3, suggesting blockage of late stage autophagy. Our results show novel functions for OGR1 in the regulation of ER stress through the IRE1α-JNK signalling pathway, as well as blockage of autophagosomal degradation. OGR1 inhibition might represent a novel therapeutic approach in IBD.

## Introduction

The two major forms of inflammatory bowel disease (IBD), Crohn’s disease and ulcerative colitis, give rise to inflammation that is linked with extracellular acidification of the mucosal tissue. In addition to inflammatory conditions, acidosis also exists in the tissue microenvironment of other pathophysiological conditions such as ischemia, tumours, metabolic, and respiratory disease^1–6^. In order to maintain pH homeostasis, cells are required to sense acidic changes in their microenvironment and respond accordingly. A family of G protein-coupled receptors (GPCRs): including ovarian cancer G-protein coupled receptor 1 (OGR1, also known as GPR68), GPR4 and T-cell death associated gene 8 (TDAG8, also known as GPR65), are activated by acidic extracellular pH. These receptors, which are almost silent at pH 7.6–7.8 and maximally active at pH 6.4–6.8^7–10^, are reported to play a role in pH homeostasis^7,11,12^, in the regulation of inflammatory and immune responses^13,14^ and in tumorigenesis^15,16^.

In several recent studies, we and others reported a link between IBD and the family of pH-sensing GPCRs^17–24^. We recently showed that IBD patients expressed higher levels of OGR1 mRNA in the mucosa than healthy control subjects^18,19^ and moreover, the deletion of OGR1 or GPR4 protects from intestinal inflammation in experimental colitis^18,20,22^. We also found that OGR1 is strongly regulated by tumour necrosis factor (TNF) via a nuclear factor (NF)-κB dependent pathway and is essential for intestinal inflammation and fibrosis^18,21^. Moreover, we previously observed that OGR1 expression is induced in human myeloid cells by TNF, PMA or LPS, whereby this effect is reversed by the c-Jun N-terminal kinase (JNK) inhibitor, SP600125, suggesting that JNK/AP1 pathway is involved in OGR1 regulation^18^. Interestingly, TDAG8, the anti-inflammatory counter-player to pro-inflammatory OGR1, has been identified as an IBD risk gene by genome wide association studies (GWAS)^25–28^. IBD-associated risk variant TDAG8 rs3742704 I231L has been described to disrupt lysosomal function, autophagy and pathogen clearance in lymphoblasts^29^. We observed that the IBD-associated risk variant TDAG8 rs8005161 presents a more severe disease course in IBD patients^23^. No biochemical changes in individuals with various genotypes of rs8005161 were observed, but we observed a lower activation of RhoA upon an acidic pH shift in IBD patients^23^. These studies suggest that TDAG8 negatively regulates inflammation in IBD; supporting the notion of an anti-inflammatory role for TDAG8^14,30,31^.

In addition to the known pro-inflammatory role of OGR1, proton-activation of OGR1 triggers Ca^2+^ release from intracellular stores, stimulates protein kinase C (PKC) signalling and activates the mitogen-activated protein kinase (MAPK), also called extracellular signal-regulated (ERK) kinase cascade^2,7,11,17,32,33^. Ca^2+^ signalling is known to play a pivotal role in ER stress^34^. Signalling through PKC is known to activate ERK^35^. MAPK/ERK signalling cascades play an important role in regulating the cellular response to various extracellular stimuli^36^. Activation occurs by sequential phosphorylation by JNK, extracellular signal regulated kinase (ERK) 1/2, p38 MAPK, ERK5, and ERK3/4^37^. We previously showed that OGR1 signalling also increased the expression of cell adhesion and extracellular matrix protein-binding genes, inflammatory response genes plus several genes linked to ER stress, e.g. activating transcription factor (ATF)3 and serpin H1, and autophagy (ATG16L1)^17^.

Importantly, acidosis is known to activate endoplasmic reticulum (ER) stress and the unfolded protein response (UPR) in numerous cell types^38–43^. Moreover, ER stress, the UPR and autophagy are critical factors contributing to IBD pathogenesis^41,44–48^. Three molecular sensors are associated with the UPR pathway, inositol-requiring enzyme 1α (IRE1α), ATF6 and PKR-like ER kinase (PERK)^49^. Under normal conditions, these ER stress sensors remain in an inactive state by coupling with binding immunoglobulin protein (BiP)^49^. Acidic activation of GPR4, another member of the pH-sensing family, which is predominately expressed in endothelial cells and only weakly expressed in other cell types^50^, stimulates all three arms of the ER stress pathways (PERK, ATF6, and IRE1α) in endothelial cells^40^.

JNK is activated in response to a wide range of stress signals, including UV irradiation, osmotic stress and hypoxia, and previous studies have linked JNK activation with tissue acidification^17,37^. Several reports indicate that ER-dependent cell death is regulated by the activation of JNK^51^, and that JNK is linked to ER stress through IRE1α^52^. We have previously shown that the human intestinal epithelial cell (IEC) line, Caco-2 overexpressing OGR1, presented pH-dependent OGR1-mediated signalling, including inositol phosphate formation, intracellular calcium/PKC, and extracellular signal-regulated kinases 1 and 2 (ERK1/2) signalling, and enhanced serum response factor (SRF)-dependent transcription under acidic pH conditions^17^. We also confirmed a several hundred-fold increased mRNA expression of OGR1 in Caco-2 cells stably overexpressing OGR1 relative to Caco-2 parental cells harbouring the empty vector (vector control (VC))^17^.

In the present study we used an OGR1-overexpressing Caco-2 cell *in vitro* model to investigate if our previously observed protective effects of OGR1 deficiency in experimental colitis are in part due to differences in UPR regulation, ER stress and autophagy.

## Results

### OGR1 induces ER stress under acidic conditions

**Figure 1:**
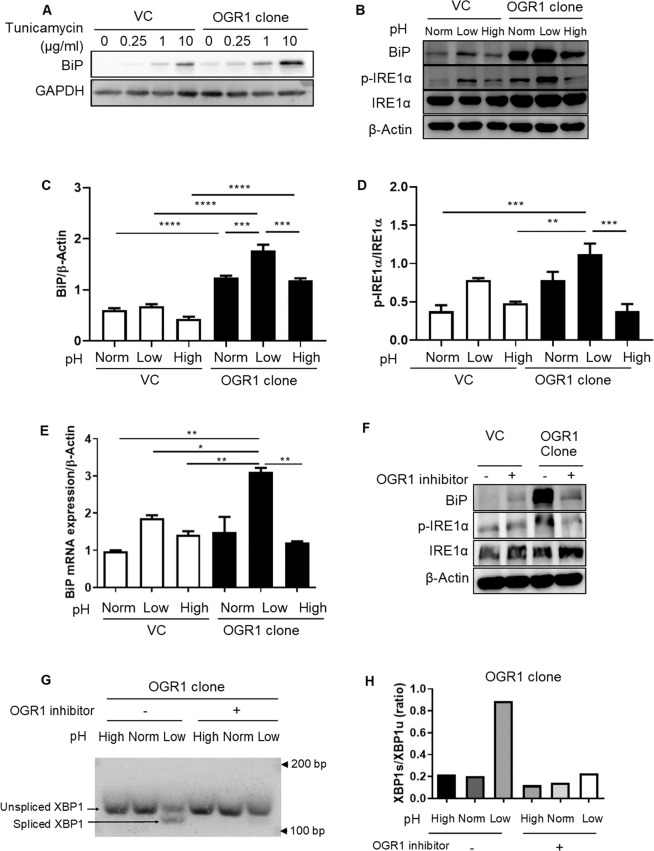
ER stress is induced by acidosis activated OGR1-mediated signalling. Caco-2 cells were subjected to different pH medium, following 4–6 h incubation in pH 7.6 serum free medium. (**A**) Vector control Caco-2 (VC) and OGR1 overexpressing Caco-2 cells where treated with tunicamycin at the indicated concentrations for 24 h. Total protein was isolated and Western blotting was performed. The results are representative of two independent experiments. (**B**) After 24 h pH shift, total protein was isolated and Western blotting was performed. The results are representative of three independent experiments. (**C**) Densitometry after normalization of BiP to β-actin and **(D)** p-IRE1α to total IRE1α. Statistical analysis was performed using one-way ANOVA followed by Tukey’s post-test. Data are presented as means ± SE of three independent experiments (*p < 0.05; **p < 0.01; ***p < 0.001; ****p < 0.0001). **(E)** After 24 h pH shift, total RNA was isolated and mRNA expression was investigated by qPCR. Statistical analysis was performed using one-way ANOVA followed by Tukey’s post-test. Data are presented as means ± SE of three independent experiments (*p < 0.05; **p < 0.01). **(F)** A specific small molecule OGR1 inhibitor (10 µM) was tested and the cells were subjected to low pH for 24 h, following 4–6 h incubation in pH 7.6 serum free medium. After 24 h pH shift, total protein was isolated and Western blot performed. Results are representative of two independent experiments. **(G)** Cells were treated as described in (**F**), then total RNA was extracted and analysed for expression of XBP1 (XBP1u) and spliced XBP1 (XBP1s) by conventional PCR. Results are representative of three independent experiments. **(H)** Quantification of the ratio of XBP1s/XBP1u was performed using ImageJ. Results are representative of two independent experiments. Statistical analysis was performed using one-way ANOVA followed by Tukey’s post-test. Data are presented as means ± SE of three independent experiments (*p < 0.05; **p < 0.01; ***p < 0.001; ****p < 0.0001). For all the panels, the experiments were repeated two to three times. pH conditions: High pH 7.5–7.8; Normal pH 7.2–7.4; Low pH 6.6–6.8.

In order to investigate the role of the pH-sensing OGR1 receptor in the induction of ER stress, OGR1-overexpressing Caco-2 and VC Caco-2 cells, were subjected to an acidic pH shift for 24 h. The stress inducer tunicamycin induced protein expression of the ER stress marker BiP in a dose dependent manner in VC Caco-2 cells and Caco-2 cells overexpressing OGR1 (Fig. 1A and Supplementary Figure 1). Acidic pH triggered the protein expression of BiP, as well as the phosphorylation of IRE1α, in Caco-2 cells overexpressing OGR1 cells (Fig. 1B and Supplementary Figure 2). Densitometry after normalization of BiP to β-actin (Fig. 1C) and p-IRE1α to total IRE1α (Fig. 1D) is presented. BiP mRNA expression also significantly increased under acidic conditions in Caco-2 overexpressing OGR1 compared to VC cells (Fig. 1E). Interestingly, at acidic pH the expression of BiP and phosphorylation of IRE1α were markedly reduced in OGR1-overexpressing Caco-2 cells in the presence of the OGR1 inhibitor (Fig. 1F and Supplementary Figure 3), suggesting that ER stress is induced by proton-activated OGR1 signalling. In OGR1 overexpressing Caco-2 cells, pH-dependent OGR1 signalling triggered the splicing of XBP1, which was prevented in the presence of the OGR1 inhibitor (Fig. 1G,H, and Supplementary Figure 4), confirming the role of OGR1 in the induction of ER stress.

### OGR1 induces ER stress via IRE1α/JNK signalling

**Figure 2:**
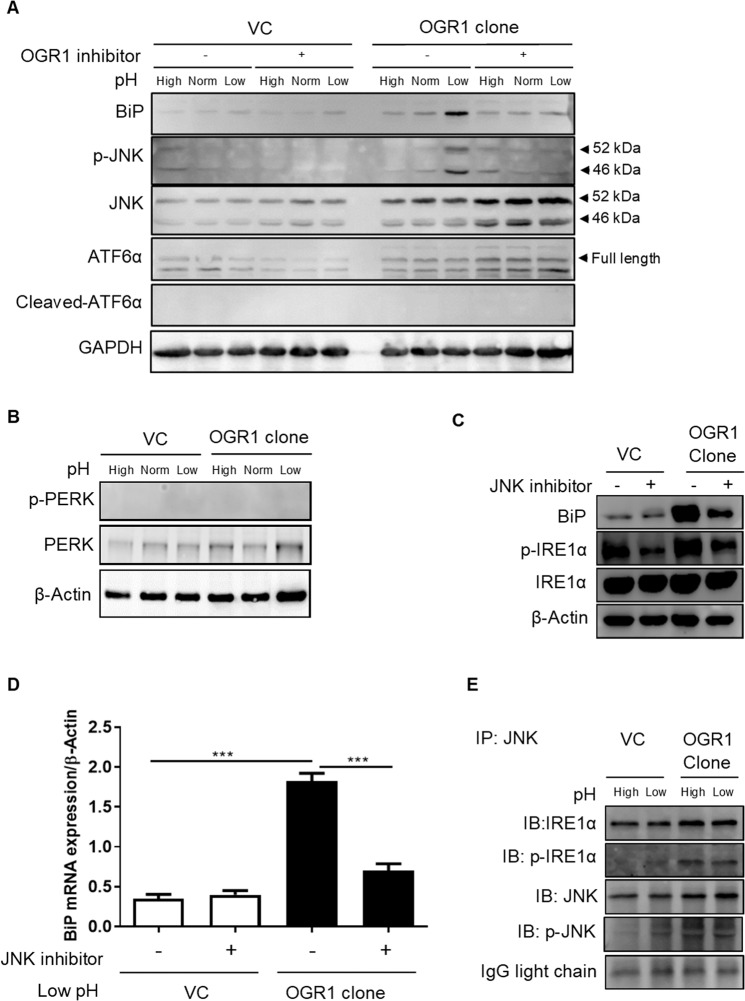
ER stress is induced by OGR1 via IRE1α/JNK signalling. **(A)** Caco-2 cells were subjected to different pH medium, with or without an OGR1 inhibitor (10 µM), following 4–6 h in pH 7.6 serum free medium. After 24 h pH shift, total protein was isolated and Western blot performed. Results are representative of two independent experiments. **(B)** Caco-2 cells were subjected to different pH medium After 24 h pH shift, total protein was isolated and Western blot performed. Results are representative of two independent experiments. **(C)** Caco-2 cells were subjected to different pH medium with or without a JNK inhibitor (10 µM) following 4–6 h in pH 7.6 serum free medium. After 24 h pH shift, total protein was isolated and Western blot performed. Results are representative of two independent experiments. **(D)** Caco-2 cells were starved and subjected to an acidic pH with or without a JNK inhibitor as described in (**C**). After 24 h pH shift, total RNA was isolated and mRNA expression was investigated by qPCR. Statistical analysis was performed using one-way ANOVA followed by Tukey’s post-test. Data are presented as means ± SE of three independent experiments (***p < 0.001). **(E)** Caco-2 cells were starved and subjected to different pH medium following 4–6 h in pH 7.6 serum free medium. After 24 h pH shift, total protein was isolated and co-IP using IRE1α antibody and JNK antibody was performed, followed by immunoblotting. Results are representative of two independent experiments. pH conditions: High pH 7.5–7.8; Normal pH 7.2–7.4; Low pH 6.6–6.8.

Next, we sought to identify the signalling factors involved in acidic pH-induced OGR1-mediated ER stress. Acidic pH induced BiP expression and JNK phosphorylation in OGR1-overexpressing Caco-2 cells compared to VC cells (Fig. 2A and Supplementary Figure 5). Importantly, BiP expression and JNK phosphorylation were prevented in the presence of the OGR1 inhibitor (Fig. 2A and Supplementary Figure 5). Strikingly, in OGR1-overexpressing cells the expression of JNK was increased in the presence of the OGR1 inhibitor. This result suggests a compensatory mechanism that would trigger JNK expression following blockade of JNK phosphorylation. Of note, acidic pH failed to induce cleavage of ATF6 (Fig. 2A and Supplementary Figure 5) or PERK phosphorylation (Fig. 2B and Supplementary Figure 6) in VC and OGR1-overexpressing Caco-2 cells. Interestingly, the JNK inhibitor reduced low pH-induced IRE1α phosphorylation (Fig. 2C and Supplementary Figure 7) and BiP mRNA expression (Fig. 2D), confirming the crucial role of JNK in OGR1-mediated induction of ER stress under acidic conditions. Moreover, Co-IP experiments showed a direct physical interaction between p-IRE1α and p-JNK in OGR1-overexpressing Caco-2 cells (Fig. 2E and Supplementary Figure 8). Results under normal pH conditions (pH = 7.2–7.4) are shown throughout the manuscript and showed no significant differences when compared with high pH (pH = 7.5–7.8) (i.e. in the expression/activation of ER stress markers or JNK (Figs. 1 and 2A)). Taken together, these results point to the notion that ER stress is induced by proton-activated OGR1-mediated signalling via the IRE1α/JNK pathway.

### Acidosis activated OGR1-mediated signalling does not induce apoptosis

**Figure 3:**
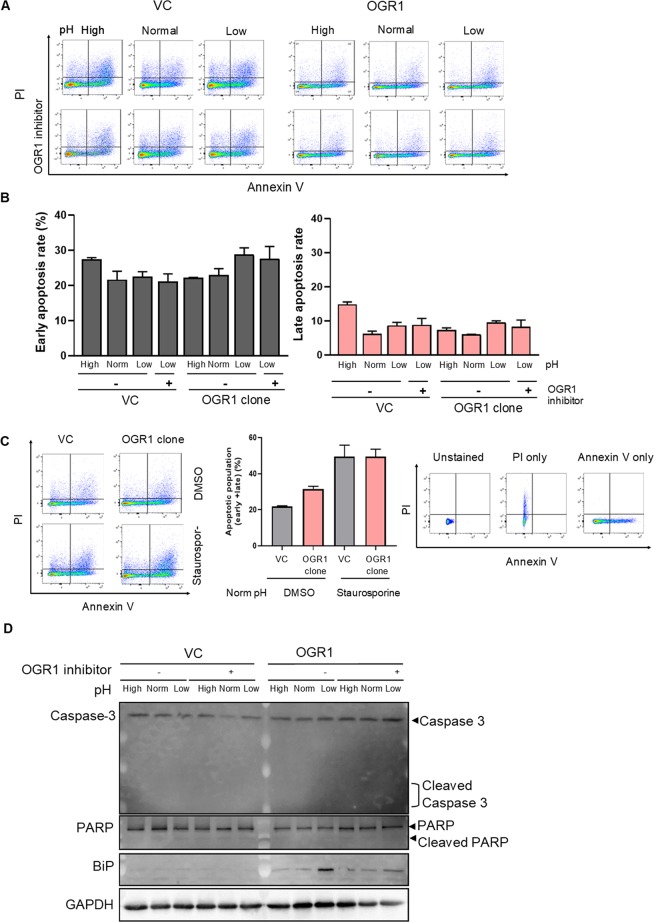
Apoptosis is not induced by acidosis activated OGR1-mediated signalling. **(A**,**B)** Caco-2 cells were subjected to different pH medium for 24 h, with or without an OGR1 inhibitor (10 µM), following 4–6 h in pH 7.6 serum free medium. Flow cytometric analysis of the percentage of annexin V-FITC and propidium iodide positive cells was performed. pH conditions: High pH 7.6–7.7; Normal pH 7.2–7.3; Low pH 6.6–6.7. Annexin V + PI- are early apoptotic cells and annexin V + PI + are late apoptotic cells. **(C)** Caco-2 cells were subjected to normal pH medium for 24 h, following 4–6 h in pH 7.6 serum free medium, with negative control (DMSO) or positive control staurosporine (1 µM) and flow cytometric analysis was performed. Staining controls; unstained or stained with either annexin V-FITC or propidium iodide. After 10 min incubation, flow cytometric analysis. Quantification was performed using FlowJo software. For all the panels, the experiments were repeated two to three times. **(D)** Caco-2 cells were treated as described for (**A**). After 24 h pH shift, total protein was isolated and Western blotting was performed. pH conditions: High pH 7.5–7.8; Normal pH 7.2–7.4; Low pH 6.6–6.8.

Since IRE1α/JNK signalling has been shown to trigger apoptosis by inhibiting Bcl-2, we investigated the impact of OGR1 activation on the induction of apoptosis. VC and OGR1-overexpressing cells where subjected to an acidic pH shift in the presence or absence of the OGR1 inhibitor for 24 h. Annexin V and PI staining followed by FACS analysis revealed that the population of apoptotic cells was not affected by the acidic pH shift in OGR1-overexpressing cells (Fig. 3A–C). Furthermore, cleavage of caspase 3 and poly (ADP-ribose) polymerase (PARP) were investigated by immunoblotting. Under the condition that BiP was upregulated on activation of OGR1, neither cleaved caspase 3 nor cleaved PARP was observed (Fig. 3D and Supplementary Figure 9), confirming that apoptosis was not induced in OGR1-overexpressing cells.

### Acidosis activated OGR1-mediated signalling blocks autophagy

**Figure 4:**
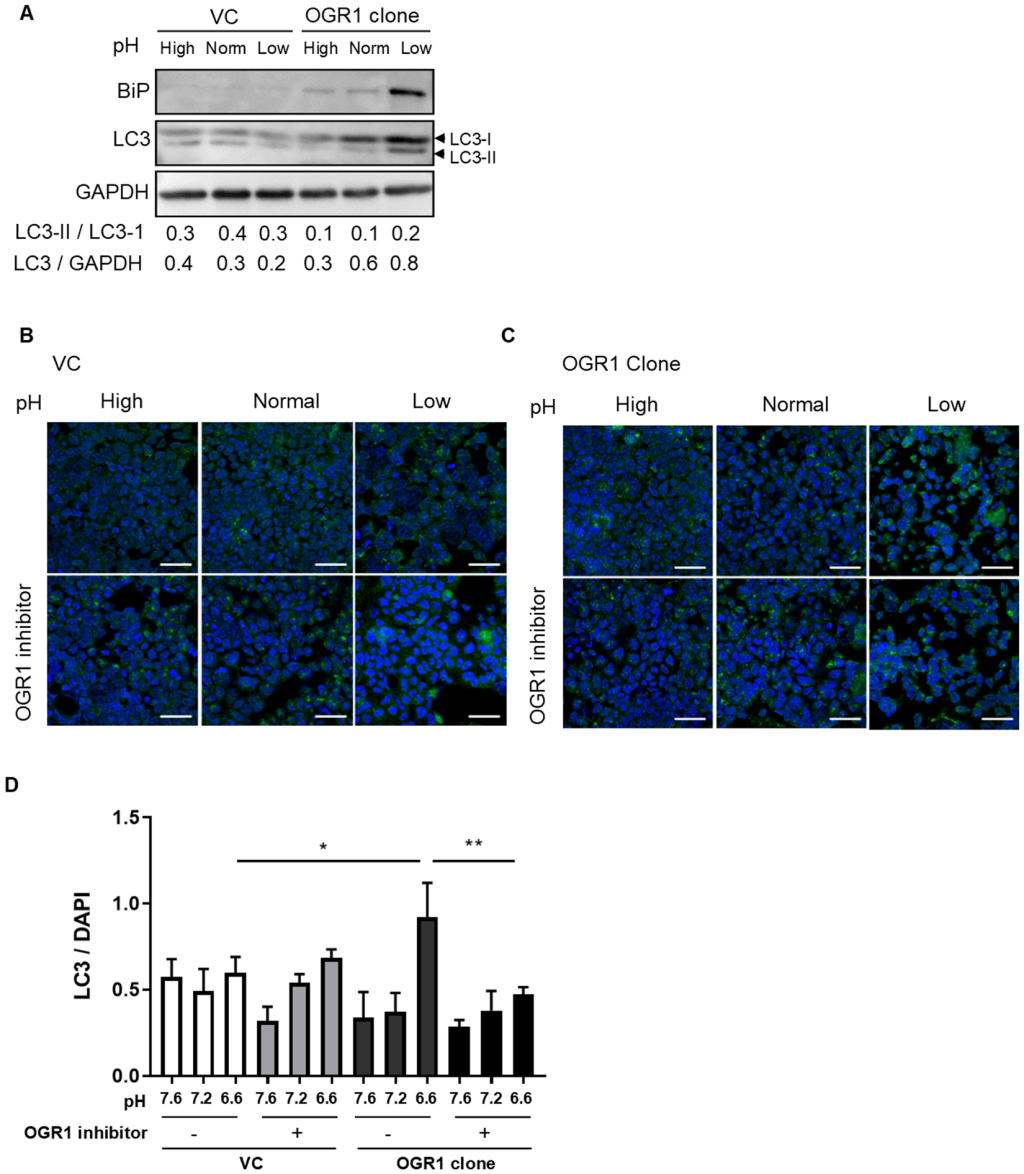
Autophagy is blocked by acidosis activated OGR1. **(A)** Caco-2 cells were subjected to different pH medium, following 4–6 h incubation in pH 7.6 serum free medium. After 24 h pH shift, total protein was isolated and Western blotting was performed. Autophagy was measured by variations in the ratio of LC3-II/LC3-I and the total amount of LC3 (LC3-I plus LC3-II) relative to GAPDH. Results are representative of two independent experiments. **(B,C)** Caco-2 cells were subjected to different pH medium, with or without OGR1 inhibitor (10 µM, following 4–6 h incubation in pH 7.6 serum free medium.) After 24 h pH shift, cells were fixed in 4% paraformaldehyde and stained with an anti-LC3 antibody. Cells were analysed by immunofluorescence microscopy and images were acquired under a confocal laser microscope. Results are representative of three independent experiments. Scale bars indicate 50 µm. **(D)** Quantification of the ratio of LC3/DAPI is presented. Changes in LC3 accumulation were calculated relative to DAPI staining from at least 4 areas. Statistical analysis was performed using one-way ANOVA followed by Tukey’s post-test. Data are presented as means ± SE of three independent experiments (*p < 0.05; **p < 0.01). pH conditions: High pH 7.5–7.8; Normal pH 7.2–7.4; Low pH 6.6–6.8.

ER stress has been linked to the blockage of autophagy. Therefore, we sought to investigate the role of OGR1 in autophagy. VC and OGR1-overexpressing Caco-2 cells were subjected to an acidic pH shift for 24 h and protein levels of LC3-I and LC3-II were investigated by immunoblotting. Acidic pH reduced the conversion of LC3-I into LC3-II, and blocked autophagosome degradation, evidenced by the accumulation of total LC3 in OGR1-overexpressing Caco-2 cells compared to VC cells (Fig. 4A and Supplementary Figure 10). We confirmed these results using immunofluorescence microscopy. OGR1-overexpressing cells subjected to an acidic pH shift showed increased LC3 staining, which was reversed in the presence of the OGR1 inhibitor. On the other hand, no changes were observed in the VC under different pH conditions with or without OGR1 inhibitor (Fig. 4B–D). These results suggested that autophagy is blocked by proton-activated OGR1 signalling.

## Discussion

**Figure 5:**
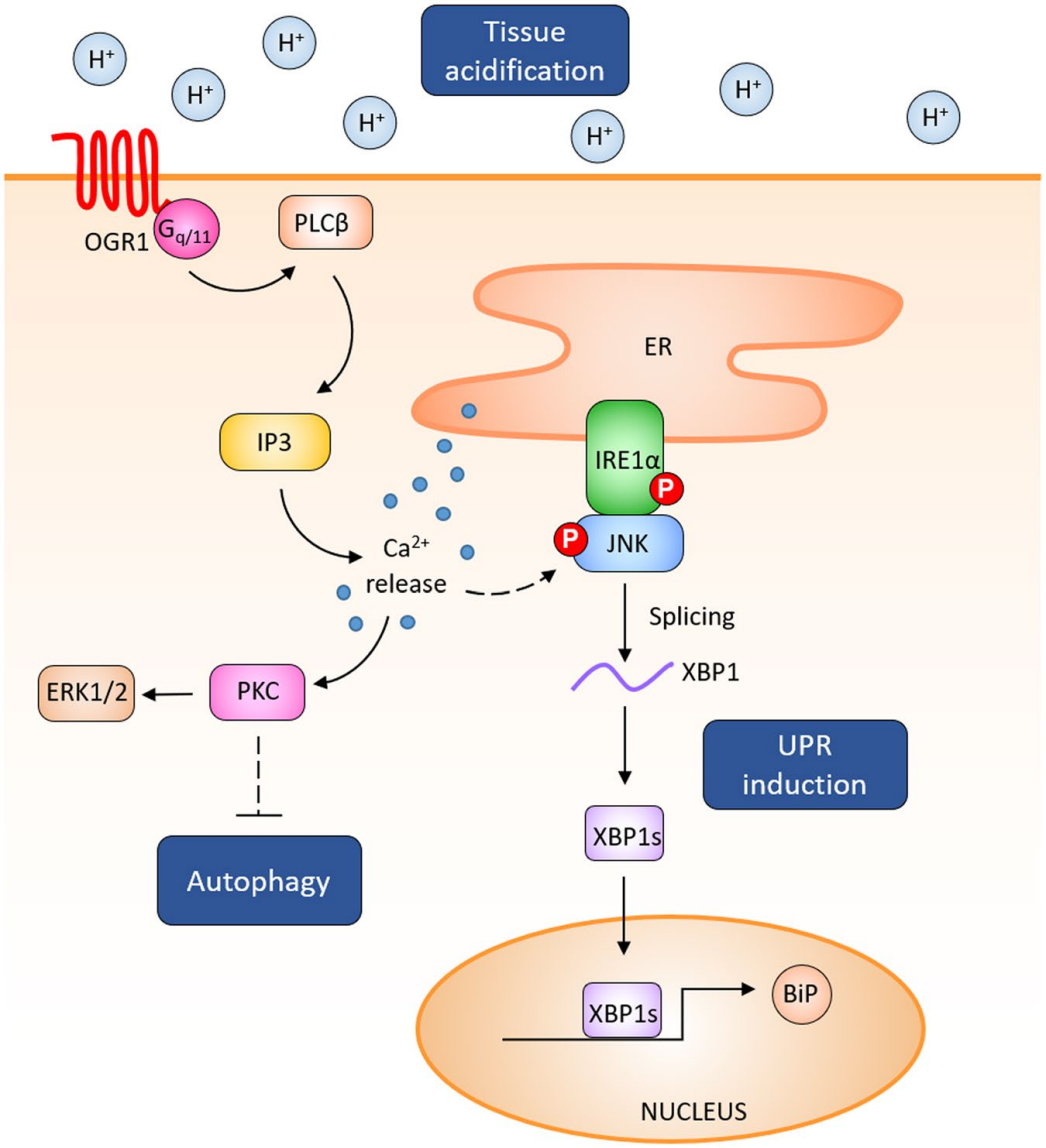
OGR1 activation triggers the expression of the ER stress marker BiP through the JNK/IRE1α signalling pathway. Following acidic activation of OGR1, JNK and the UPR molecule IRE1α are phosphorylated and induce downstream XBP1 splicing, which in turn leads to the expression of the ER stress marker BiP in IECs. Acidic activation of OGR1 leads to the blockage of late stage autophagy.

Our results show that proton-activated OGR1-mediated signalling triggers the expression of the ER stress marker BiP together with the phosphorylation of IRE1α and splicing of XBP1 in a human intestinal epithelial cell line stably overexpressing OGR1. Furthermore, we found that activation of OGR1 triggers the IRE1α-JNK signalling pathway, but not the other branches involved in the UPR, namely PERK or ATF6. Acidosis and activation of the UPR in intestinal epithelial cells are closely linked to the development of intestinal inflammation (Fig. 5). Our results provide confirmatory evidence of a crucial role for OGR1-mediated IRE1α/JNK activation in the induction of ER stress under low pH conditions, which might underlie the reported impact of OGR1 in the development of IBD^18,19^. In our previous studies, we observed significant and pH-dependent OGR1-mediated signalling, including IP3/Ca^2+^/ERK signalling and enhanced SRF transcription under acidic pH conditions (pH = 6.8)^17^.

The link between acidic activation of GPCRs and MAPKs has been long established. Several reports have demonstrated that GPCRs can induce intracellular signal transduction through ERK1/2 and MAPK pathways^53,54^. Acidic OGR1 stimulation has been shown to trigger IL-6 expression through ERK1/2 and p38 activation in human airway smooth muscle cells^30^. Proton-dependent Ca^2+^ release from intracellular stores has been shown to trigger the MEK/ERK1/2 pathway, thereby linking acidification with cell proliferation^2^. Recent reports have shown that ER stress triggers apoptosis via the activation of the IRE1α-JNK signalling pathway^55,56^. Surprisingly, we did not detect apoptotic processes following acidic activation of the IRE1α-JNK pathway, suggesting that OGR1-mediated IRE1α-JNK signalling may therefore promote cell survival together with OGR1 inflammatory signalling in intestinal epithelial cells. Interestingly, the pro-apoptotic role of JNK has been suggested to be strongly influenced by the parallel activation of cell survival pathways and the strength of the apoptotic response. Several reports indicate that while the sustained activation of JNK is associated with apoptosis, the acute and transient activation of JNK is crucial for cell proliferation and survival^57–59^. In this regard, several studies have also suggested that two functionally distinct phases of JNK signalling are involved in the ER stress response, an early phase that promotes survival and a late phase associated with cell death. Brown *et al*. showed that early JNK activation in ER-stressed cells triggers the expression of several apoptosis inhibitors early in the ER stress response. Using MEFs from IREα- and TRAF2-deficient mice, these authors showed that the early JNK activation requires both IRE1α and TRAF2^60^.

Additionally, acidic activation of OGR1 has been suggested to enhance survival in osteoclasts through the induction of PKC activation, which may affect the phosphorylation of pro- or anti-apoptotic proteins, or stimulate ERK1/2 signalling^61,62^. Although the role of PKC in autophagy regulation is still controversial, several studies have suggested that PKC is involved in the suppression of autophagy^63^. In HEK293 cells stably expressing LC3, activation of PKC significantly attenuated autophagy induced by starvation or rapamycin through the phosphorylation of LC3, while inhibition of PKC with pharmacological inhibitors increased autophagy^64^. PKC has also been shown to mediate cisplatin nephrotoxicity *in vivo* by suppressing autophagy^49^. Moreover, PKC has also been suggested to block autophagy in pancreatic ductal carcinoma cells through the activation of tissue transglutaminase 2^65,66^.

The expression of OGR1 is strongly upregulated in ischemic myocardium and has been associated with survival in cardiomyocytes^67^, as well as the induction of neurogenesis in mice^68^. Studies in primary prostate tumours derived from OGR1-expressing cells showed that OGR1-mediated signalling pathways did not affect growth or apoptosis in primary tumors^69^. However, in endplate chondrocytes proton activated OGR1-mediated Ca^2+^ flux from intracellular stores led to apoptosis^70^.

Acidic activation of OGR1 triggers the activation of JNK-mediated ER stress, which suggests a role of IRE1-JNK signalling in controlling autophagy^71^. Strikingly, our results show an increase in total LC3 accumulation, but not in LC3-I to LC3-II conversion in OGR1 overexpressing cells following acidic pH shift, indicating an OGR1/IRE1/JNK-mediated blockage of the final stages of autophagy. The role of IRE1α-JNK in regulating autophagy remains a matter of controversy. Notably, JNK has been shown to play a role in autophagy suppression in neurons^72^. Conversely, the activation of ER stress triggered both apoptosis and autophagy through the IRE1/JNK/beclin-1 axis in breast cancer cells^73^. Another study showed that IRE1α upregulated autophagy under ER stress independently of XBP1 signalling^71^. Recently, phosphorylation of the anti-apoptotic protein BCL-2 by IRE1α was linked to the initiation of autophagy through the modulation of the activity of Beclin-1^74^, an essential component of the autophagy machinery^72,75,76^. JNK has been shown to participate in the expression of MAP1LC3 following TNF stimulation in vascular smooth muscle cells^77^. Inhibition of the JNK pathway blocked ceramide-induced autophagy and up-regulation of LC3 expression^78^. Xie *et al*. reported that JNK plays a crucial role in bufalin-induced autophagy in HT-29 and Caco-2 cells^79^.

In our hands, acidic activation of OGR1 in an OGR1-overexpressing cell model increased accumulation of LC3, but not the conversion of LC3-I into LC3-II, pointing to a blockage of late stage autophagy. Of note, our results suggest that partial activation of OGR1 under normal pH conditions is able to block late-stage autophagy in OGR1 overexpressing cells, and this effect is enhanced when OGR1 is fully activated at low pH. Interestingly, ROS-induced JNK activation induces both autophagy and apoptosis in cancer cells^80^. Taken together, our results suggest that acidic activation of OGR1 triggers opposite pathways leading to cell survival as well as the blockage of the late stages of autophagy. It is plausible that acidic activation of OGR1 initiates autophagy through IRE1α-JNK signalling together with parallel signals that block autophagosomal degradation, thereby contributing to the pro-survival and pro-inflammatory effects of OGR1.

Further investigations are required to elucidate the exact mechanisms of OGR1/IRE1/JNK-mediated blockage of the late stages of autophagy. Taken together, our results indicate that OGR1 may have novel functions in the regulation of ER stress and autophagy and could represent a novel therapeutic target of IBD.

## Methods

### Reagents

All chemicals were obtained from Sigma-Aldrich (St. Louis, MO, USA), including Tunicamycin (T7765) and Staurosporine (S6942), unless otherwise stated. A specific c-Jun N-terminal kinase (JNK) inhibitor (SP600125) was purchased from Calbiochem (La Jolla, CA). The OGR1 inhibitor was kindly provided by Takeda Pharmaceuticals San Diego, USA. All cell culture reagents were obtained from Thermo Fisher (Allschwil, Switzerland), unless otherwise specified.

### Cell culture and pH shift

Caco-2 cells (LGC Promochem, Molsheim, Switzerland) and derived clones stably overexpressing OGR1 were cultured in a humidified atmosphere with 5% CO_2_ at 37 °C in Dulbecco’s Modified Eagle’s Medium (DMEM) with GlutaMAX (Invitrogen, Carlsbad, CA USA) supplemented with 400 µg/ml geneticin (G418)-selective antibiotic (Invitrogen) and 10% fetal bovine serum (Invitrogen). Construction of the hu-OGR1-pcDNA3.1 + plasmid, clone generation, selection and characterization has been previously described^17^.

### pH treatment

pH shift experiments were carried out in serum-free RPMI-1640 medium supplemented with 2 mM GlutaMAX and 20 mM HEPES (all from Invitrogen). For pH adjustment of the RPMI medium, the appropriate quantities of NaOH or HCl were added, and the medium was allowed to equilibrate in the 5% CO_2_ incubator at 37 °C for at least 36 h before it was used. Caco-2 cells were seeded and cultured for 24–48 hours before the pH shift was performed. Cells were starved for 4–6 h in serum free RPMI medium, pH 7.6, and then subjected to an acidic pH shift for 24 h.

### Western blotting and Co-immunoprecipitation

Following treatment, the cells were lysed with ice-cold Mammalian protein extraction reagent (M-PER, Thermo Fisher Scientific, Reinach, Switzerland). The following antibodies were used: BiP (Cat. No. 3177; Cell Signalling Technology, Danvers, MA, USA), phospho-IRE1α (Cat. No. NB100-2323, Novus Biologicals, Littleton, CO, USA), IRE1α (Cat. No. 3294, Cell Signalling Technology), phospho-PERK (Cat. No. 3179S, Cell Signalling Technology), PERK (Cat. No. 3192S, Cell Signalling Technology), phospho-JNK (Cat. No. 9251, Cell Signalling Technology), JNK (Cat. No. 9252, Cell Signalling Technology), ATF6α (Cat. No. sc-166659, Santa Cruz, CA, USA), LC3 (Cat. No. L7543; Sigma-Aldrich), Caspase 3 (Cat. No. 9662, Cell Signalling Technology), PARP (Cat. No. 9542; Cell Signalling Technology) and GAPDH (Cat. No. MCA4740, BIO RAD Hercules, CA, USA). Primary antibodies were used at 1:1000 dilution for Western blotting.

Co-immunoprecipitation (Co-IP) was performed overnight at 4 °C using the IRE1α antibody (Cat. No. 3294, Cell Signalling Technology) and JNK antibody (Cat. No. 9251, Cell Signalling Technology) at 1:200 dilution. Immunocomplexes were collected with protein G sepharose beads (17-0618-01, GE Healthcare, Glattbrugg, Switzerland) for 30 min at 4 °C prior to Western blotting. Densitometry of bands was measured using ImageJ software.

### Immunocytochemistry

Cells were washed with PBS and fixed in 4% paraformaldehyde for 15 min at 4 °C and then permeabilized in 100% methanol (Sigma-Aldrich) for 10 min. After blocking with 3% bovine serum albumin (BSA), cells were incubated with LC3 antibody (Cat No. 2992, Cell Signalling Technology) at 1:200 dilution overnight at 4 °C. Cells were then incubated with an Alexa Fluor 488-conjugated anti-rabbit antibody (Cat. No. A11032, Invitrogen) for 1 h and DAPI (Sigma-Aldrich) for 5 min before mounting with anti-fade medium (Dako, Glostrup, Denmark). Cells were analysed by a Leica SP5 laser scanning confocal microscope (Leica Microsystems, Wetzlar, Germany). Fluorescence images were processed using Leica confocal software (LAS-AF Lite, Leica Microsystems). Quantification of LC3/DAPI was performed using ImageJ software [National Institutes of Health]^81^ using the software’s colour threshold tool, which calculates the area of positive staining. The resulting value was normalised to quantification of nucleus staining and represents the positively stained area normalised to cell numbers present in the given area.

### Annexin V staining

Externalization of phosphatidylserine in apoptotic cells was detected with Annexin V and dead cells were stained with propidium iodide (PI), using the Dead Cell Apoptosis Kit (Annexin V FITC and PI, Cat. No. V13242, Thermo Fischer Scientific), according to the manufacturer’s instructions. After 10 min incubation at room temperature in the dark, cells were washed in PBS and resuspended in the binding buffer. Single-cell suspensions were analysed by FACS-Canto II flow cytometry (BD Biosciences, Allschwil, Switzerland) using FlowJo software.

### RNA extraction and real-time quantitative PCR (qPCR)

Total RNA was isolated using the RNeasy Mini Kit (Qiagen, Hombrechtikon, Switzerland) according to the manufacturers’ instructions. For removal of residual DNA, a DNase treatment was performed, according to the manufacturer’s instructions, for 15 min at room temperature. For reverse transcription, the High-Capacity cDNA Reverse Transcription Kit (Applied Biosystems, Foster City, CA, USA) was used following the manufacturer’s instructions. Determination of mRNA expression was performed by qPCR on a 7900HT real-time PCR system (Applied Biosystems) under the following cycling conditions: 20 s at 95 °C, then 45 cycles of 95 °C for 1 s, and 60 °C for 20 s with the TaqMan Fast Universal Master Mix. Samples were analysed as triplicates. Relative mRNA expression was determined the by the ΔΔCt method, which calculates the quantity of the target sequences relative to the endogenous control β-actin and a reference sample. TaqMan Gene Expression Assays (all from Applied Biosystems), used in this study were human BiP (Hs 00268858-S1) and human β-actin Vic TAMRA (4310881E).

### XBP1 splicing assay

XBP1 splicing was measured by specific primers flanking the splicing site yielding PCR product sizes of 152 and 126 bp for unspliced XBP1 and spliced XBP1 mRNA, respectively. Primers (forward 5′-CCTGGTTGCTGAAGAGGAGG-3′, reverse 5′-CCATGGGGAGATGTTCTGGAG-3′) were used. PCR was carried out at 95 °C for 15 min, then 40 cycles at 94 °C for 30 sec, 56.5 °C for 30 sec, and 72 °C for 1 min. The size difference between the spliced and the unspliced XBP1 is 26 nucleotides. These products were resolved on 3.5% agarose gels. Band intensity of XBP1s and XBP1u was determined using ImageJ and the ratio of XBP1s/XBP1u was quantified.

### Statistical analysis

Statistical analyses were performed using GraphPad Prism 8 (GraphPad Software, San Diego, CA). Data are presented as means ± SE and statistical significance was determined using the Kruskal-Wallis test. p < 0.05 was considered significant. Where indicated, one-way ANOVA was performed, followed by Tukey’s post hoc test.

## Supplementary information

Supplementary information

## Data Availability

The datasets generated during and/or analysed during the current study are available from the corresponding author on reasonable request.
