# Supplementary material for: Activation of pH-Sensing Receptor OGR1 (GPR68) Induces ER Stress Via the IRE1α/JNK Pathway in an Intestinal Epithelial Cell Model
Source: Sci Rep. 2020 Jan 29;10:1438. doi: 10.1038/s41598-020-57657-9 (PMC6989664; doi:10.1038/s41598-020-57657-9)
Supplement: Supplementary file 1 — Supplementary information [file 41598_2020_57657_MOESM1_ESM.pdf]

Supplemental Material for Manuscript

ACTIVATION OF pH-SENSING RECEPTOR OGR1 (GPR68) INDUCES ER STRESS VIA THE IRE1α/JNK PATHWAY IN AN INTESTINAL EPITHELIAL CELL MODEL

Chiaki Maeyashiki[1*], Hassan Melhem[1*], Larissa Hering[1], Katharina Baebler[1], Jesus Cosin-Roger[1], Fabian Schefer[1], Bruce Weder[1], Martin Hausmann[1], Michael Scharl[1,2], Gerhard Rogler[1,2], Cheryl de Valliere[1§], Pedro A. Ruiz[1§]

A

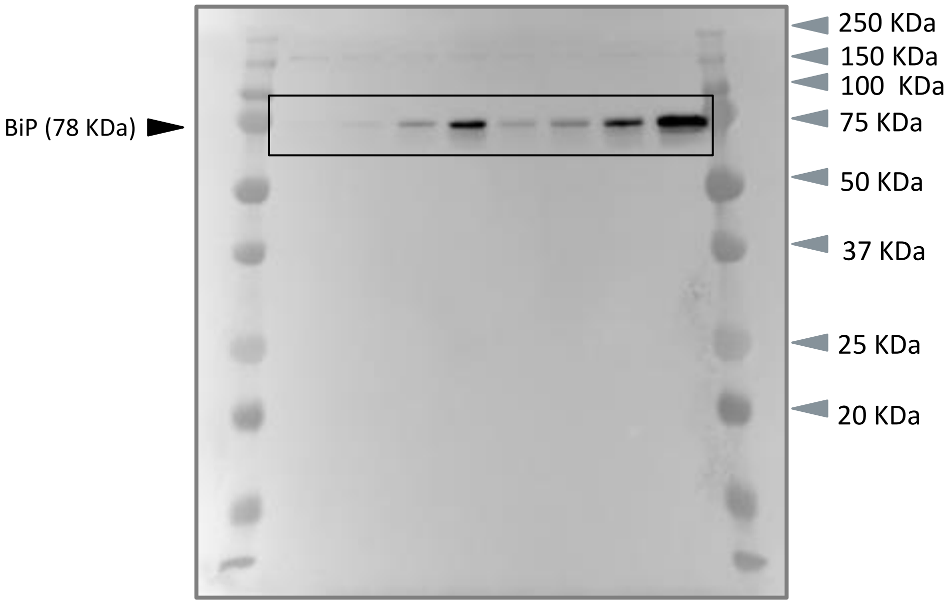

B

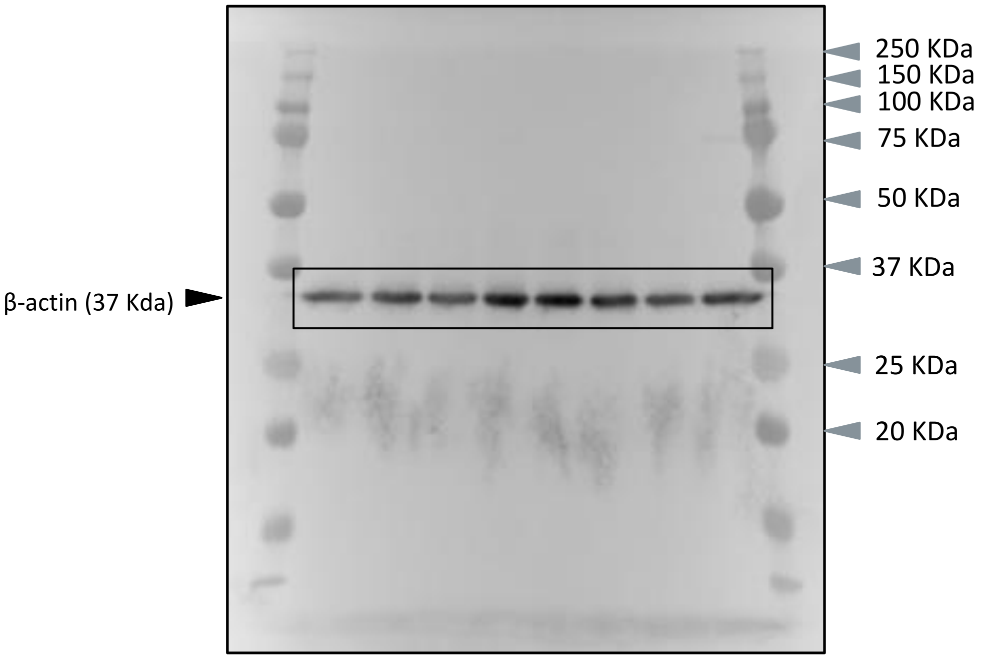

Supplementary Figure 1. Uncropped versions of blots shown in Figure 1A.

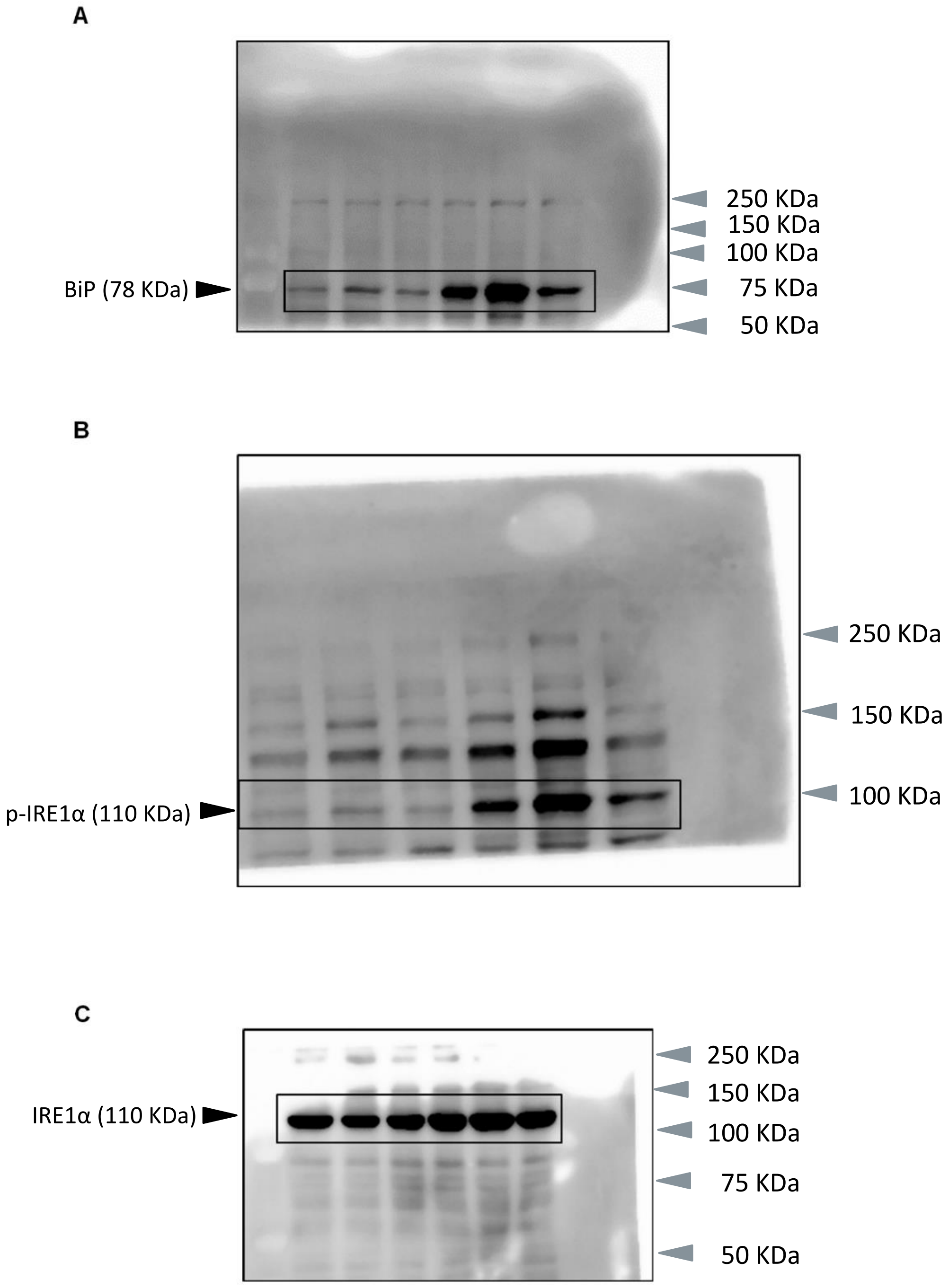

Supplementary Figure 2. Uncropped versions of blots shown in Figure 1B.

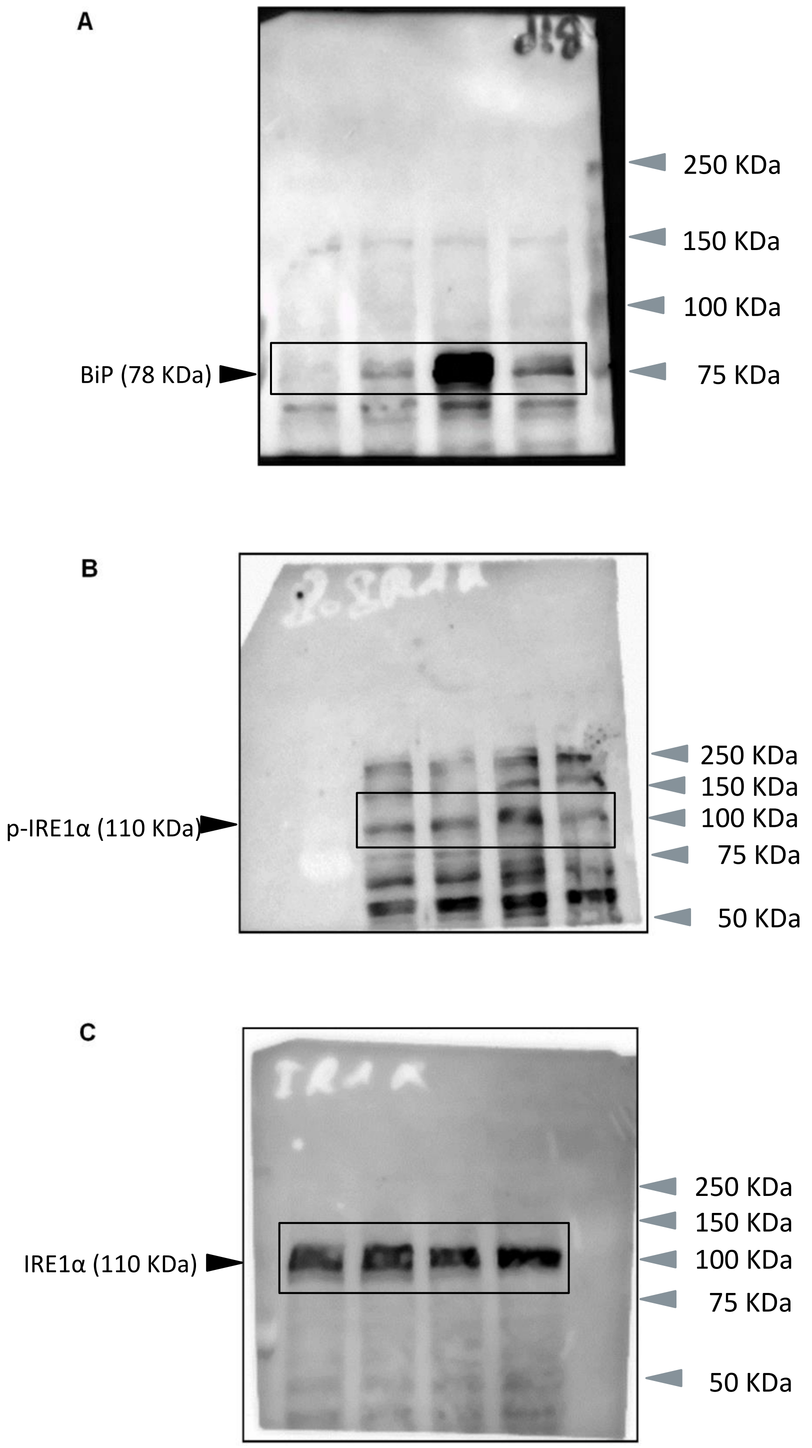

Supplementary Figure 3. Uncropped versions of blots shown in Figure 1F.

A

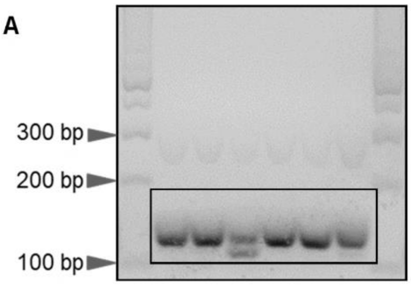

Supplementary Figure 4. Uncropped versions of blots shown in Figure 1G.

A

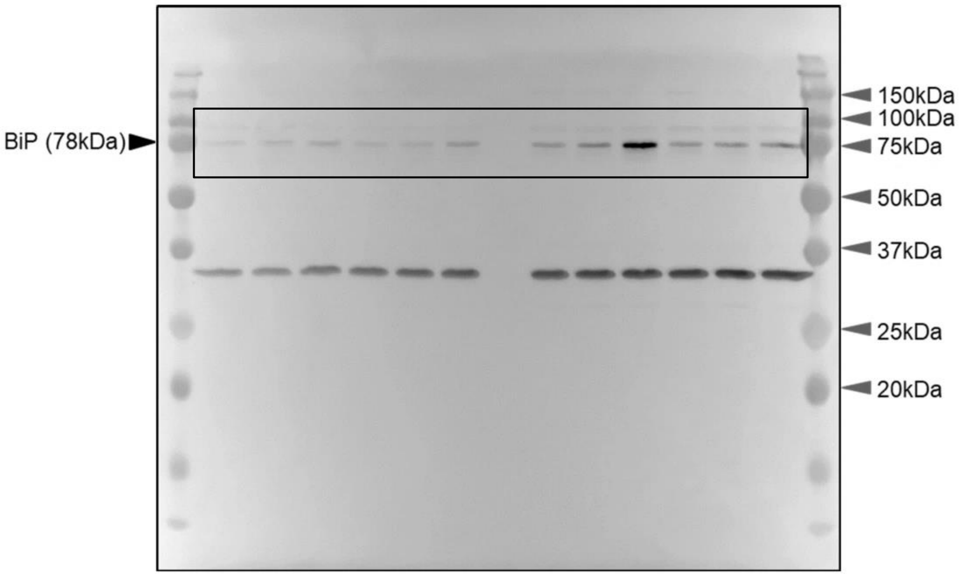

Supplementary Figure 5. Uncropped versions of blots shown in Figure 2A.

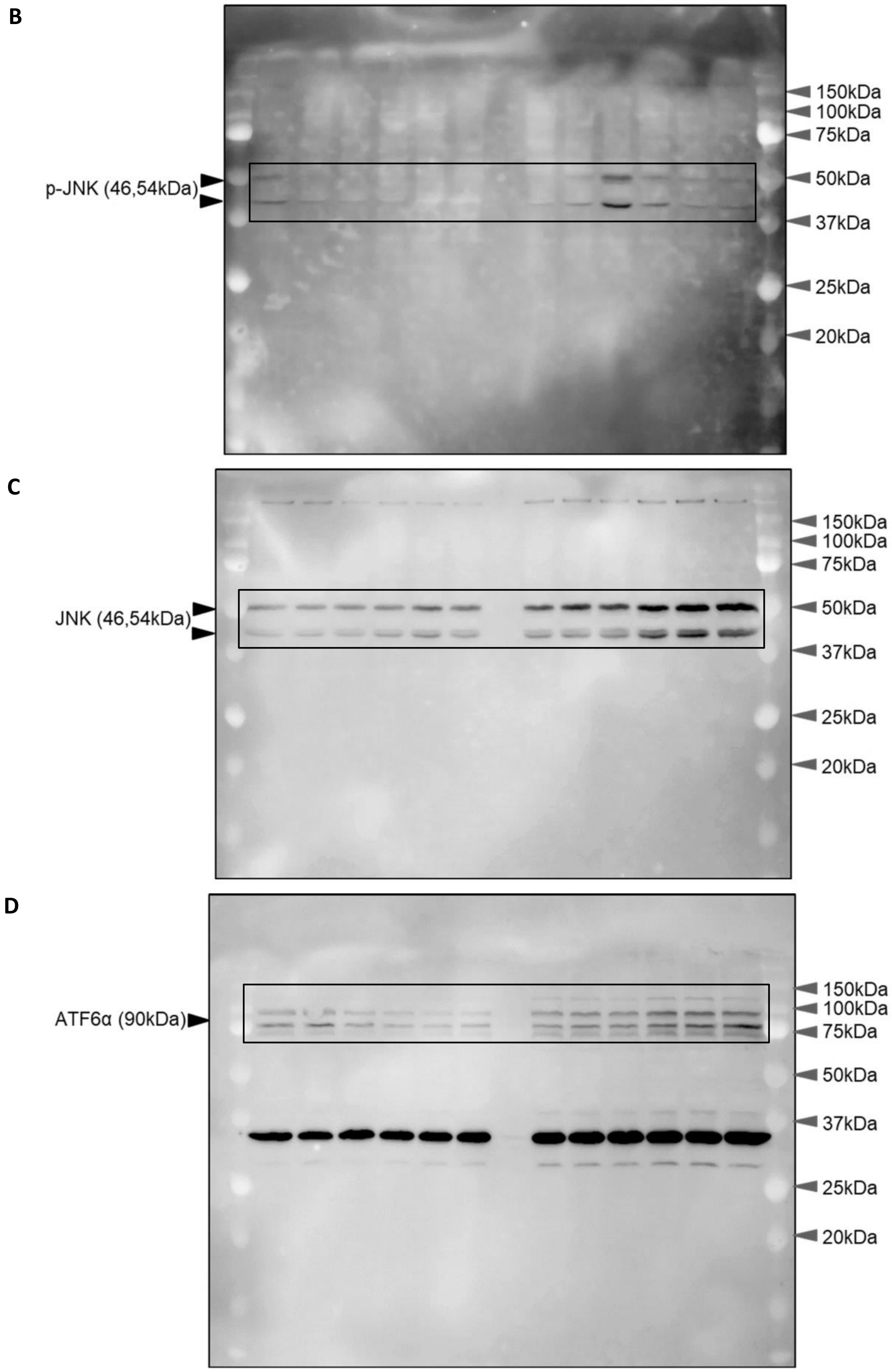

Supplementary Figure 5. Uncropped versions of blots shown in Figure 2A.

E

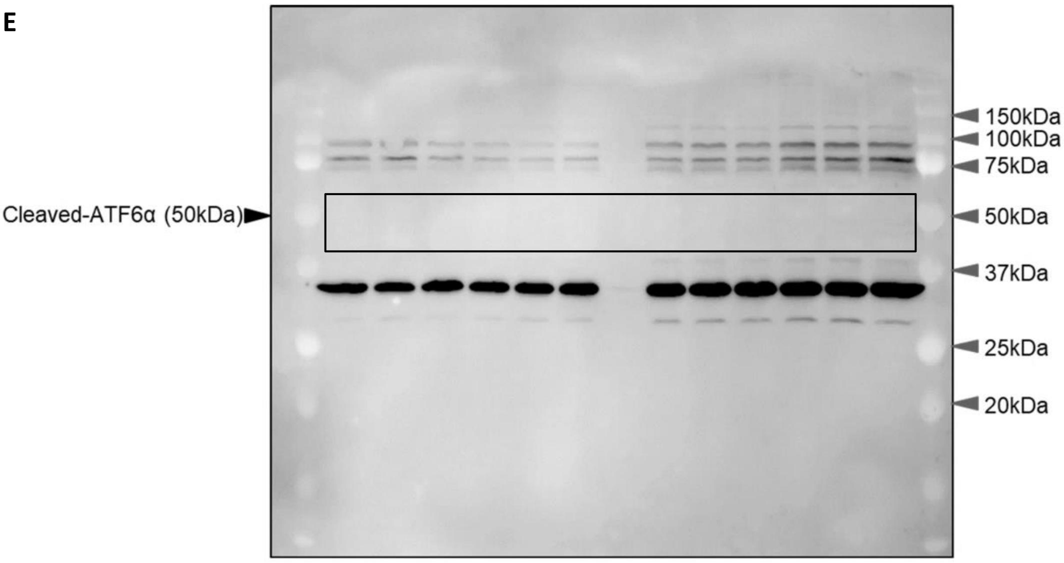

F

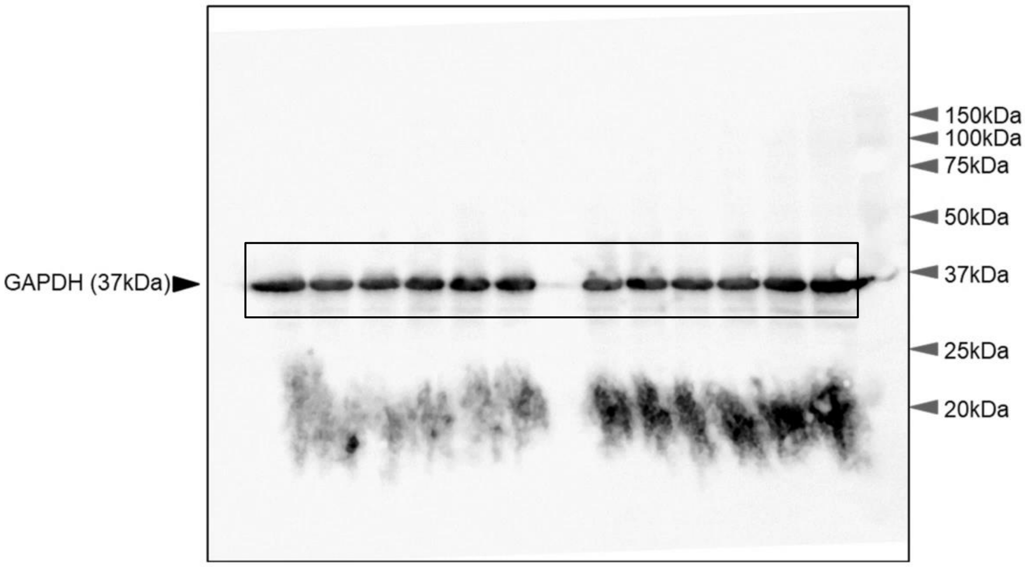

Supplementary Figure 5. Uncropped versions of blots shown in Figure 2A.

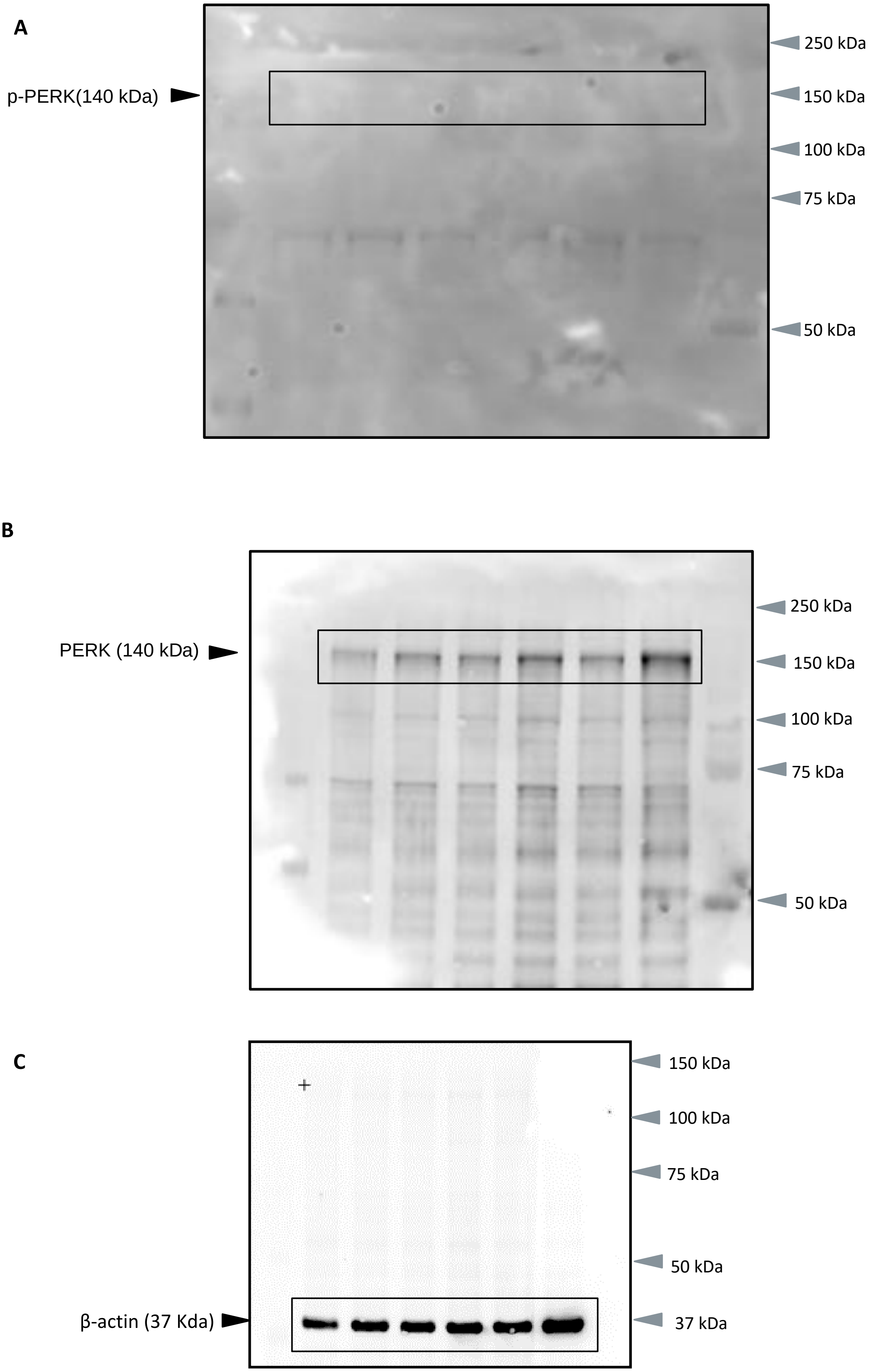

Supplementary Figure 6. Uncropped versions of blots shown in Figure 2B.

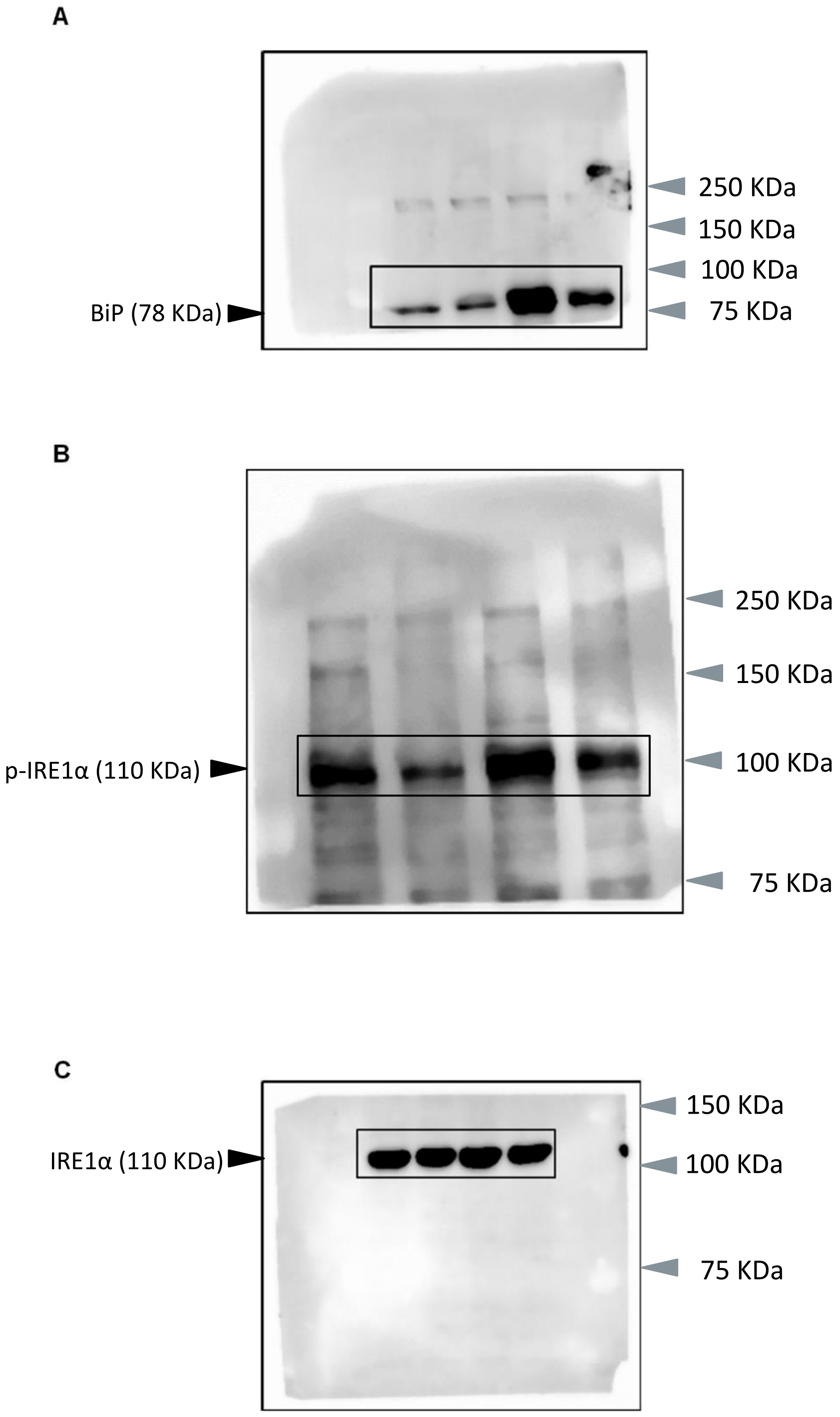

Supplementary Figure 7. Uncropped versions of blots shown in Figure 2C.

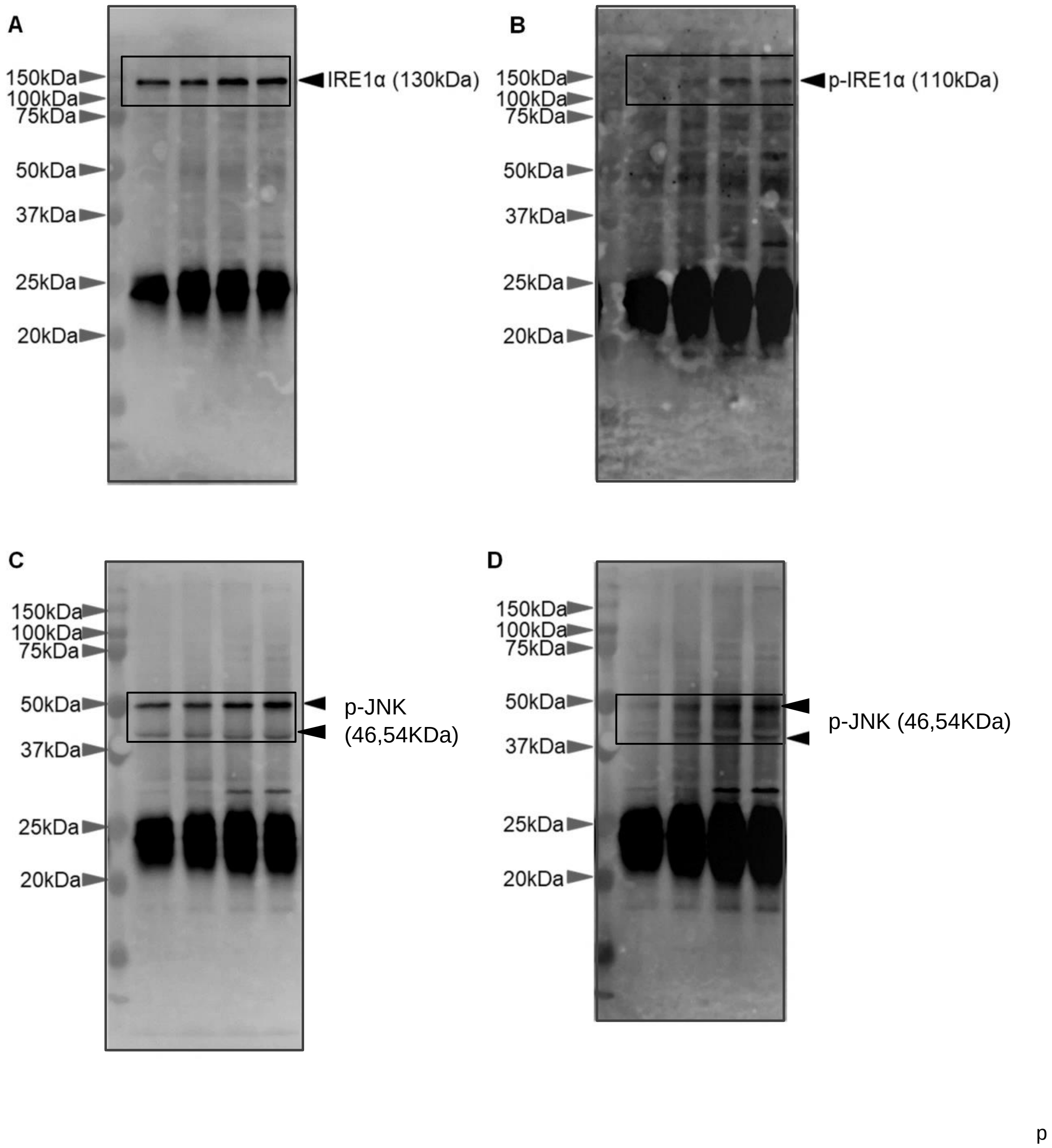

Supplementary Figure 8. Uncropped versions of blots shown in Figure 2E.

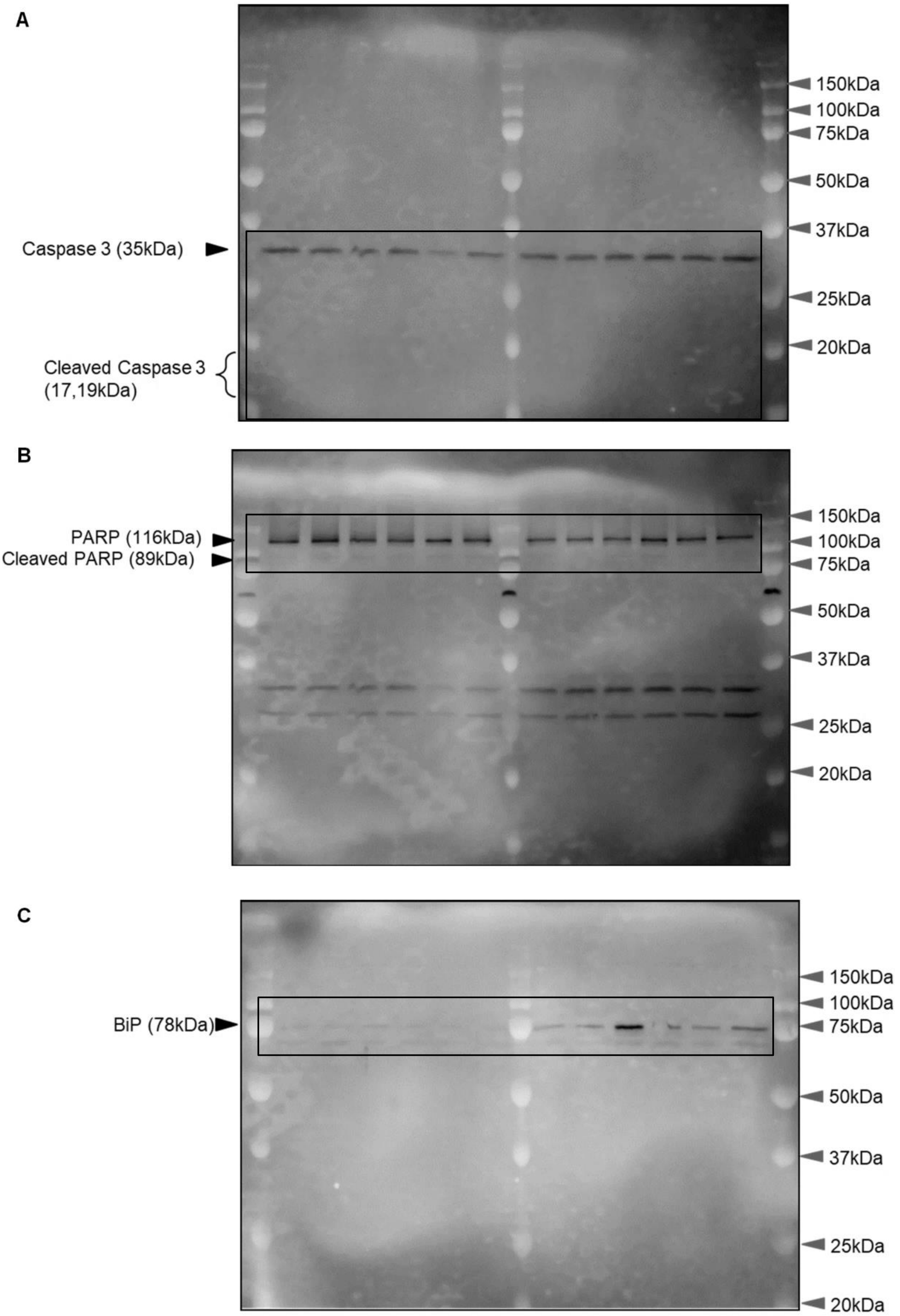

Supplementary Figure 9. Uncropped versions of blots shown in Figure 3D.

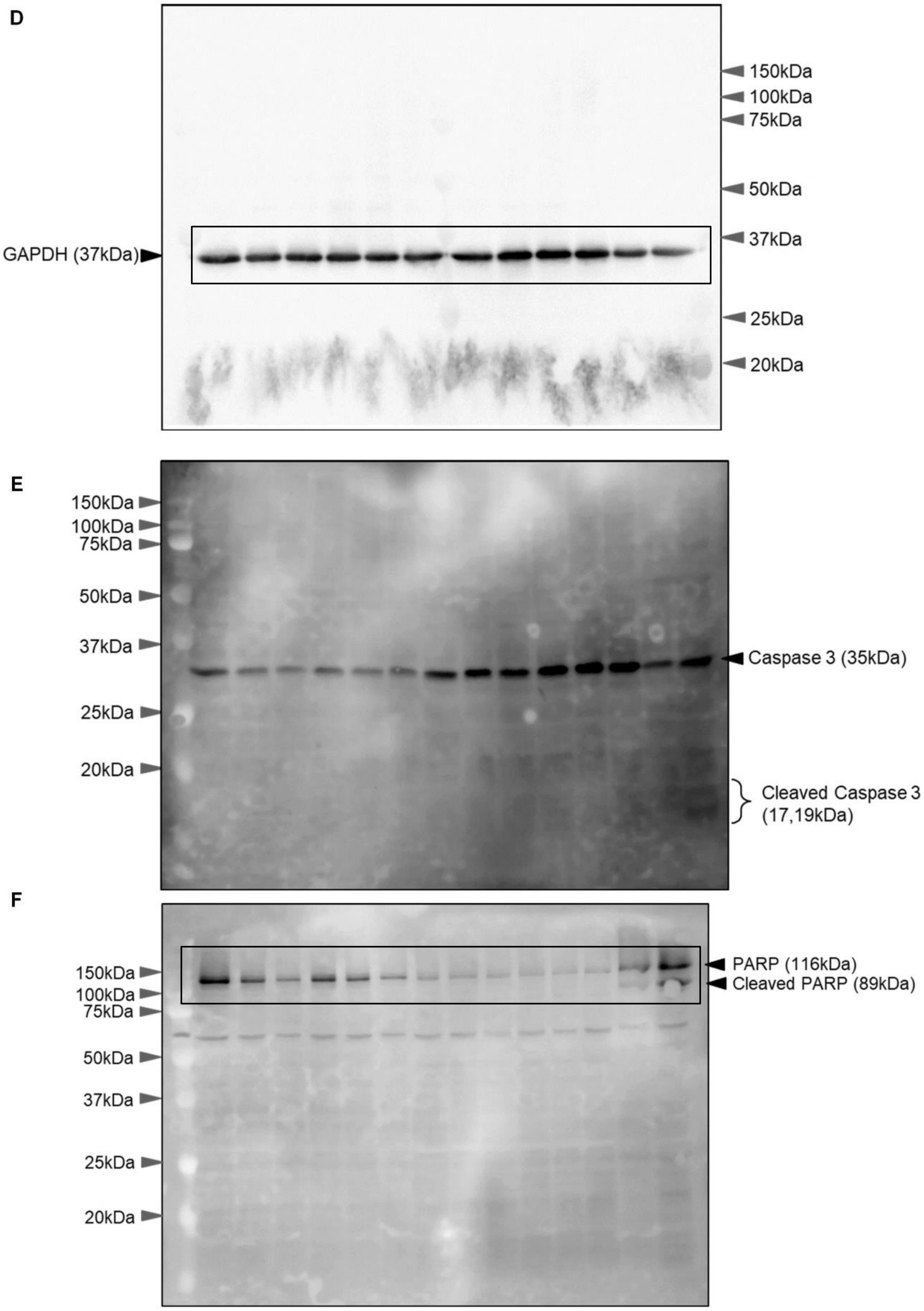

Supplementary Figure 9. Uncropped versions of blots shown in Figure 3D.

G

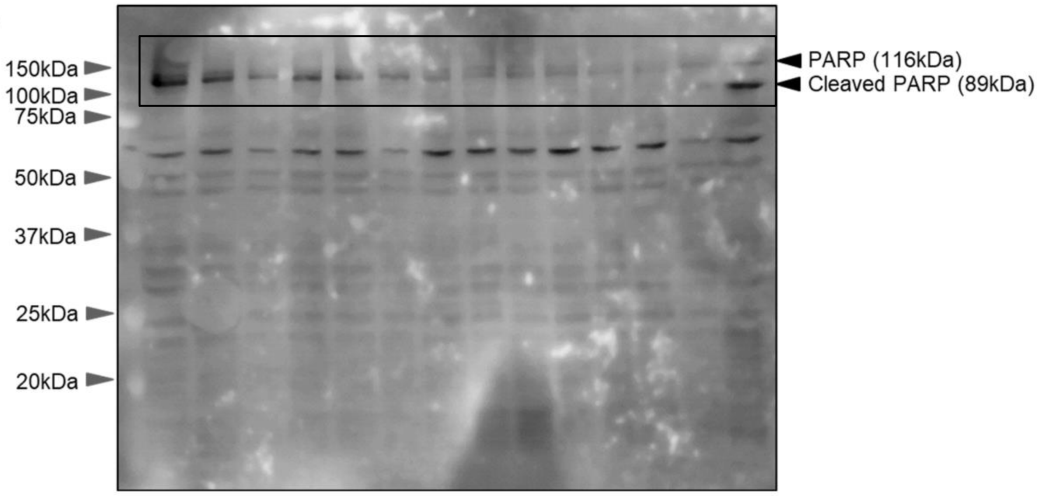

H

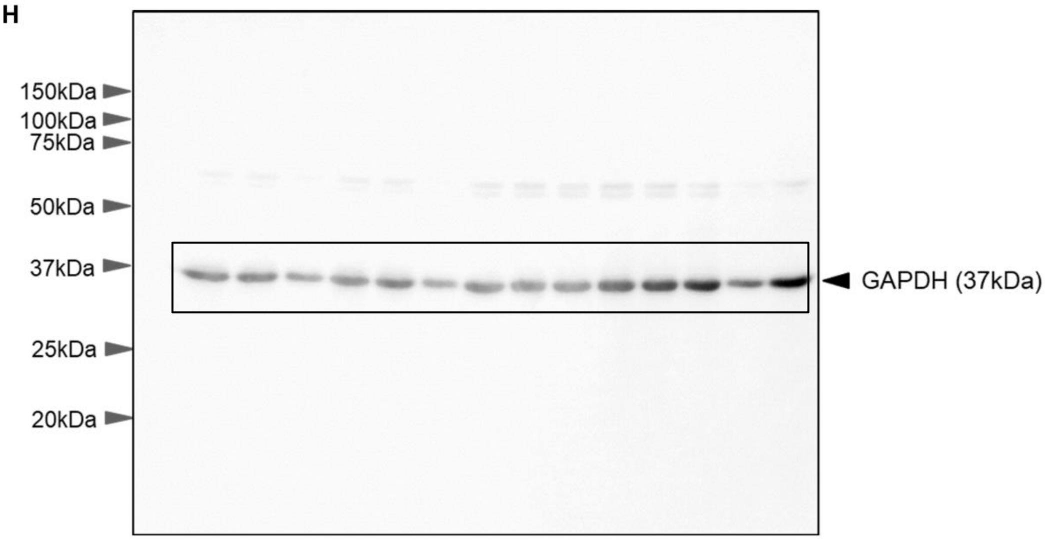

Supplementary Figure 9. Uncropped versions of blots shown in Figure 3D.

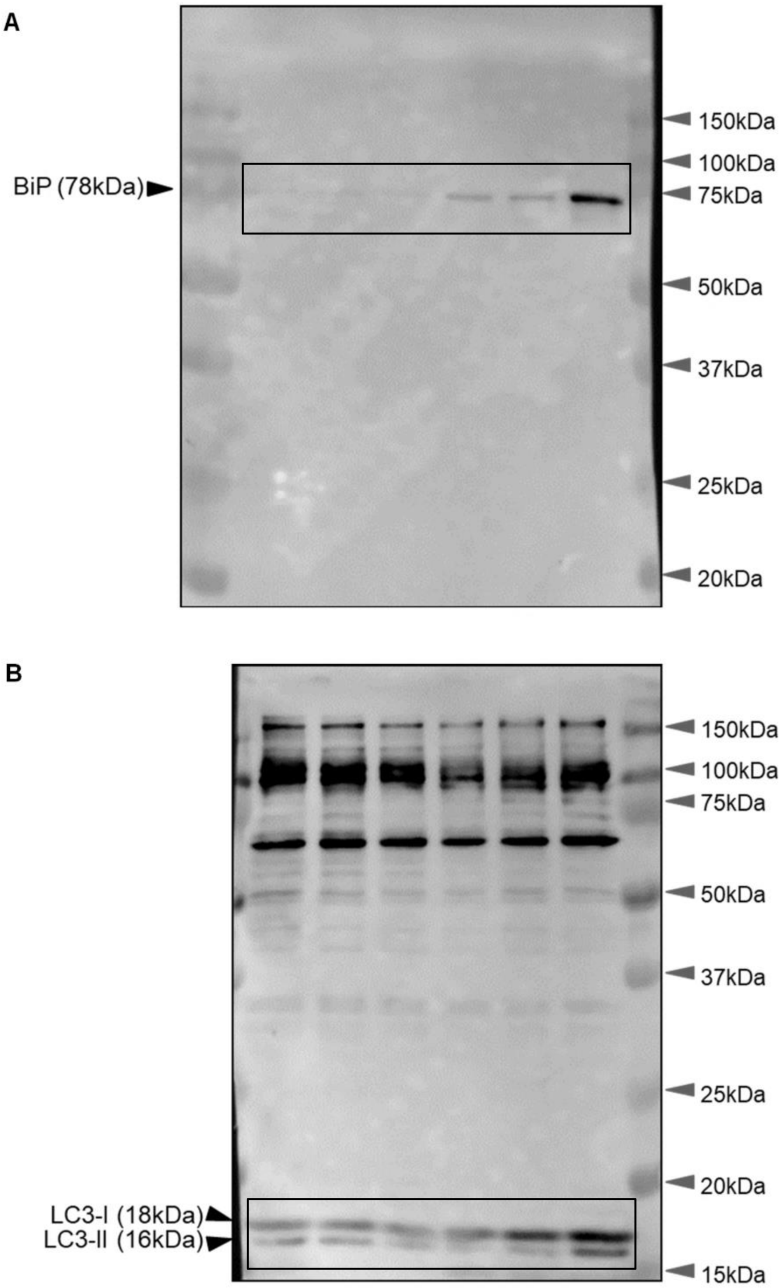

Supplementary Figure 10. Uncropped versions of blots shown in Figure 4A.

C

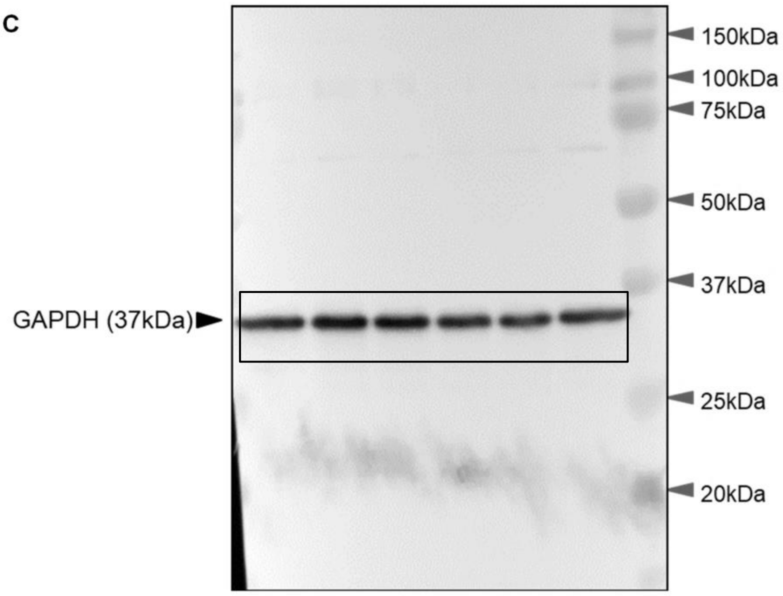

Supplementary Figure 10. Uncropped versions of blots shown in Figure 4A.
